# Neuroscience on breaking bad news: Effects of physicians’ response on patient emotion and trust

**DOI:** 10.3389/fpsyg.2022.1006695

**Published:** 2022-10-17

**Authors:** Yan Song, Yifan Xiu, Wei Li, Fang Wang

**Affiliations:** ^1^School of Business and Management, Shanghai International Studies University, Shanghai, China; ^2^Oncology Department, Jinzhou Central Hospital, Jinzhou Medical University, Jin Zhou, China

**Keywords:** breaking bad news, EEG experiment, patient emotion, physician’s response, video-mediated, time-frequency analysis, communication strategy

## Abstract

**Background:**

The outbreak of COVID-19, due to restrictions on patients’ access to hospitals, makes patient mental health a severe problem to solve, especially for cancer patients. Delivering bad news has become one of the abilities that physicians need to improve. Former research has proposed communication strategies like SPIKES to respond to patients’ emotions. However, existing strategies lack systematic and structural responses to different cues and concerns of patients.

**Objective:**

This study aims to investigate whether and how the response styles of information delivery, empathy, and authority affect patient emotions and trust in order to present a structural response system. Furthermore, we explore the correlation between strategies and EEG markers to moderate emotions and trust.

**Methods:**

This research selects different scenarios and strategies in the context of breast cancer and performs two experiments. First, we performed a behavioral experiment with 93 medical students and 15 breast cancer patients. Moreover, an EEG experiment with 53 students *via* video stimuli was conducted to explore the moderate function between strategies and emotions/trust. We use time-frequency analysis and the repeated measure ANOVA method to explore the association between strategy and EEG components. Furthermore, we perform a GLM method to investigate the relationship between EEG components and patient emotion and trust.

**Results:**

For the first time, this study proposes the strategy matrix. The response strategies NPIm and NRIa play important roles in this system. In behavioral experiments, information delivery, empathy, and authority strategy significantly affect emotions and trust. The scenario is significant as a moderator. In the EEG experiment, strategy NPIm has more correlation with parietal alpha power than other strategies, and parietal alpha power has a significant effect on emotions, which verifies that empathy-related cerebral activities affect emotions and trust.

**Conclusion:**

According to the strategy matrix, physicians could apply strategy ERIa in most scenarios, and strategy NRIa in many scenarios, which means information provision is significant when it comes to responding to patients’ cues and concerns. The most important strategy that physicians need to avoid is the authority strategy. Refusing to respond to patients’ cues and concerns may cause their dislike. Moreover, through the EEG experiment, we verify that empathy affects emotions and trust from a neuroscience perspective and propose parietal alpha and frontal alpha as neuro-markers to moderate emotions and trust. Physicians could adjust strategies through these EEG markers.

## Introduction

With the outbreak of the COVID-19, due to restrictions on patients’ access to hospitals, doctors cannot communicate face-to-face with patients, making patient mental health a severe problem to solve, especially for cancer patients.

Many kinds of bad news can damage cancer patients’ mental health. Delivering bad news has become one of the abilities that physicians need to improve. Bad news is defined as any news that significantly and negatively alters the patient’s view of their future (Buckman, 1984). Life-altering news can be associated with emotions like shock, fright, sadness (Schofield et al., 2003), or reactions of avoidance and denial (Vos and de Haes, 2007).

The last decade has witnessed significant progress in the domain of breaking bad news. As one of the most difficult and demanding tasks that oncologists face, delivering bad news has lots of difficulties. Researchers have explored and created communication strategies. In terms of patient emotional improvement, strategies like SPIKES focus on the effect of empathy response (Atienza-Carrasco et al., 2018; Toutin-Dias et al., 2018; Marschollek et al., 2019; Mirza et al., 2019; Almaiman et al., 2020; Rasmus et al., 2020; Bukowski et al., 2021). Apart from that, the information need (Friedrichsen et al., 2000; Almyroudi et al., 2011) plays an important role in patient satisfaction and decision-making (Friedrichsen et al., 2000; Almyroudi et al., 2011). The doctor’s authority also has a significant affection for patient satisfaction. Ting et al. (2016) argued that patients desired a partnership with the doctor. The responses to the decision-making and information dispensation statements were distinctively doctor-centered (Ting et al., 2016). However, little research focuses on the effects of these factors on patient emotions in the breaking bad news area. In terms of patient trust, recent research argues that responding to the patient’s emotions effectively will increase their trust in the doctor and promote their wellness (Ong et al., 2000; Fallowfield and Jenkins, 2004; Blanch-Hartigan, 2013; Johnson and Panagioti, 2018). Based on recent research, whether and how the response styles of information delivery, empathy, and authority affect patient emotions and trust has become an important question.

In the field of doctor-patient communication, most of the measurement methods of dependent variables (emotion, trust, etc.) use questionnaires to investigate the feelings of patients (Omne-Ponten et al., 1994; Hjerl et al., 2003; Arndt et al., 2005; Kumin Seo, 2010; Zwingmann et al., 2017), but there are problems with the questionnaire method: Participants do not know what their complex feelings are. Participants can not clearly describe those feelings. Participants do not want to reveal their true thoughts in the questionnaire. Which makes it difficult to measure patients’ real immediate emotional responses and deliver bad news. EEG measurements could explain the relationship between the effects of response styles and emotion/trust from a neuroscience standpoint.

Lots of literature investigates the correlation between EEG activity and behavior. In terms of information processing, Schutter and van Honk (2006) stated that frontal midline theta activity is related to the cognitive aspect of human behavior (Schutter and van Honk, 2006; Arnau et al., 2021) and that theta activity is also associated with working memory, action selection in the medial prefrontal cortex (Ishii et al., 1999; Başar-Eroglu and Demiralp, 2001; Kubota et al., 2001; Onton et al., 2005; Wang et al., 2005; Tsujimoto et al., 2006), and feedback processing (Cohen et al., 2007). Liang et al. (2021) found that nonlinear amplitude modulation in mid-frontal beta oscillation correlated with people’s working memory capacity. In terms of emotion processing, Heller and Nitscke (1997) introduced asymmetries of parietal alpha power, indicating arousal and intensity of emotional processing (Heller and Nitscke, 1997). Besides that, frontal alpha is related to emotion processing (Schmidt and Trainor, 2001; Ohme et al., 2009; Vecchiato et al., 2010). Positive emotion-related emotions were processed in left frontal brain areas, whereas negative emotions engaged right frontal brain regions. Yan et al. (2021) suggested that frontal alpha asymmetry had correlation with emotions. Similarly, Yang et al. (2021) stated that Frontal EEG asymmetry is an effective biomarker of emotional reactivity and regulation. Yang et al. (2020) discovered that network connections in the high Gamma band are associated with significant differences in pleasant, neutral, and negative emotional states (Yang et al., 2020). Gannouni et al. (2021) use EEG signals to detect emotion and find the relevant electrodes for an arbitrarily subject for every emotional state in every frequency band (Gannouni et al., 2021). In terms of trust, Wu et al. (2021) revealed that in repeated interactions, decisions to trust strangers (i.e., identification-based trust) activated the ventral striatum (Wu et al., 2021). Oh et al. (2022) state that frontal lobes in the alpha wave have active connectivity in trust. Through EEG measurements, we could verify the function of strategies from a neuroscience perspective.

Emotion recognition methods based on physiological signals mainly include emotion recognition based on the autonomic nervous system (ANS) and the central nervous system (CNS). Besides EEG method of emotion recognition, researchers tend to apply other physiological signals like electrocardiogram (ECG), electromyogram (EMG), galvanic skin response (GSR), respiration (RSP), etc. (Shu et al., 2018). The physiological signals are in response to the CNS and the ANS of the human body, in which emotion changes according to Connon’s theory (Cannon, 1927). There are attempts of applying ANS activity in health care area, Guo et al. (2013) present a pervasive and unobtrusive system for sensing human emotions based on Galvanic Skin Response (GSR) signal from human bodies.

Against this background, the principal aim of this study is fourfold. First, to test the effects of response strategies (information delivery, empathy, and authority) on emotions and trust in hearing bad news. Second, to test the medical scenario as a moderator to investigate which strategy is effective or avoidable in a particular scenario. Third, to test whether and how these strategies affect emotions and trust through EEG activities and find related EEG markers. Fourth, to verify the third aim through investigating the correlation between EEG markers and emotions and trust.

## Materials and methods

### Study design

In this study, we conducted a behavioral experiment (93 medical students and 15 breast cancer patients) and an EEG experiment (53 students) to investigate whether and how the response styles of information delivery, empathy, and authority affect patient emotion and trust under the background of breaking bad news. We also looked into the moderating effect of medical scenarios and the association between strategy and EEG components. In order to accomplish the investigation above, we conducted a 6 (Scenario: Doubt, Fear of Death, Fear of the Future, Surgery, Hair Loss, Upper Limb Disorders) x 5 (Strategy: NRIa, NPIm, ERIa, ERAb, EPAEm) within-subject design for both experiments. Strategy NRIa and ERIa represent information delivery styles, NPIm and EPAEm represent empathy styles, and ERAb is represented for authority. Six scenarios were presented as “Doubt,” “Fear of Death,” “Fear of the Future,” “Surgery,” “Hair Loss,” and “Upper Limb Disorders,” ranging from consultation, treatment, and “back to society” stage. The intervention materials were texts (for behavioral experiments) and videos (for the EEG experiment) with six versions of a bad-news consultation. Participants were asked to read patient lines, and physicians’ responses and indicate their feelings.

### Materials

#### Intervention materials for behavioral experiments

We produced texts with six versions of a bad-news consultation. They showed an encounter between an experienced senior oncologist and a standardized patient receiving bad news in six different scenarios. The response of the oncologist to the emotional cues or concerns of the patient differed in each version. According to Verona Coding Definitions of Emotional Sequences (VR-CoDES; Zimmermann et al., 2011), which details the classification of expression of patient emotion and clinicians’ response, we presented the physicians’ response communication strategies in these experiments: Non-Explicit Reduce-Space Information advise (NRIa), Non Explicit Provide-Space Implicit empathy (NPIm), Explicit-Reduce Space Information advise (ERIa), Explicit Reduce-Space Active blocking (ERAb), Explicit Provide-Space empathy (EPAEm; Table 1).

**Table 1:** Communication strategy description based on VR-CoDES.

NO.	Code	Response rules	Definition
1	NRIa	Non-Explicit-Reduce Space-Information-advise	When physicians provide information or give advice, they do not involve patients cues and concerns, which can be coded as “NRIa.”
2	NPIm	Non-Explicit-Provide Space-Implicit-empathy	Provide space through empathy for further exploration response. It does not explicitly ask for further clarification or explicitly mention the nature and emotions of the cues and concerns.
3	ERIa	Explicit-Reduce Space-Information-advise	ERIa is a clear response to cues and concerns. It provides information or advice. The response clearly recognizes the cues/concern, but does not further explore it.
4	ERAb	Explicit-Reduce Space-Active-blocking	The response clearly refuse to communicate to the patient’s cues/concerns, and is accompanied by denial, rejection or derogation of the patient’s expression. These responses may be based on facts or emotions. It also includes statements that weaken or disagree with the patient’s cues and concerns
5	EPAEm	Explicit-Provide- Space-empathy	Physicians will rationalize or share the patient’s emotions, which may or may not accompany the physicians’ own feelings. The patient’s emotions will definitely be mentioned, and emotion-related content will also be involved.

In terms of medical scenarios, they are based on the causes of psychological distress. There are several factors that relate to psychological distress. (1) Uncertainty of disease. (2) self-image. (3) role function (Krigel et al., 2014).

When it comes to the uncertainty of the disease, Patients are lacking information about their disease and treatment trajectory, which makes them fear deterioration and death.

In terms of self-image, breast surgery and hair loss can make patients question the concept of integrity. Cohen et al. found that women who had BCS experienced significantly greater psychological distress and marginally worse Quality of Life 40 months after surgery (Ohen et al., 2000). Chemotherapy-induced alopecia has effects on the quality of life among breast cancer patients. Hair loss consistently ranks amongst the most troublesome side effects, is described as distressing, and may affect the body image (Lemieux et al., 2008).

In terms of role function, many patients describe a loss or frustration at no longer being able to maintain previous roles (a healthy person) or sustain a full load at work or marriage. So they may experience distress and shock when it comes to changing their role, facing the future, and being ready to return to society. Women with upper extremity lymphedema had high levels of psychological distress, and high levels of sexual, functional, and social dysfunction (Passik et al., 1995).

To wrap it up, six scenarios were presented as “Doubt,” “Fear of Death,” “Fear of the Future,” “Surgery,” “Hair Loss,” and “Upper Limb Disorders,” ranging from consultation, treatment, and “back to society” stage. The descriptions of the scenarios and patient cues and concerns are shown in Table 2. More detailed information is shown in appendix Table 1.

**Table 2:** Description of scenarios and patient cues and concerns.

Scenario	Description	Patients’ expression
doubt	Patient has doubts about the result he/she received,thinking the result is not his/her own.	Sir! That cannot be true! This is not My report, it’s someone else’s!
Fear of death	After patient accepts the fact of having cancer, he/she thinks cancers are terminal.	I do not know if I will die because of it, I cannot even think about it.
Fear of future	After patient accepts the fact of having cancer, he/she fears that cancer may ruin his/her marriage or relationship.	I’m not married yet, what should I do in the future?
Surgery	In the course of treatment, conservative treatment is useless, cancer cells begin to spread, and A total mastectomy is required.	I cannot face a mastectomy. After my breasts are gone, my body shape has changed, am I still a woman? At least not a complete woman.
Hair loss	Due to chemotherapy or other reasons, patient has symptoms of alopecia.	If my hair is gone, how can I go outside? Even if I survive the cancer, how can I go to work without my hair?
Upper limb disorders	Upper limb disorders caused by operations can make life more difficult.	If I cannot move my arm, even cannot comb my hair, I would rather die than be disabled.

#### Intervention materials for the EEG experiment

The dialogue context was filmed in videos, a total of 30 videos. Each video lasted 13 s. Videos take patients from the first-person point of view. The context of the video was physicians’ response to patients’ emotions. The background of the video was a real hospital scene, the physician role in the video was played by Dr. Li Wei, chief physician of the oncology department of Jinzhou Central Hospital, Liaoning Province, who had extensive clinical experience. All materials were revised by her in order to restore the real doctor-patient communication scenario to a great extent. The video screenshot is shown in appendix Figure 1.

**Figure 1:**
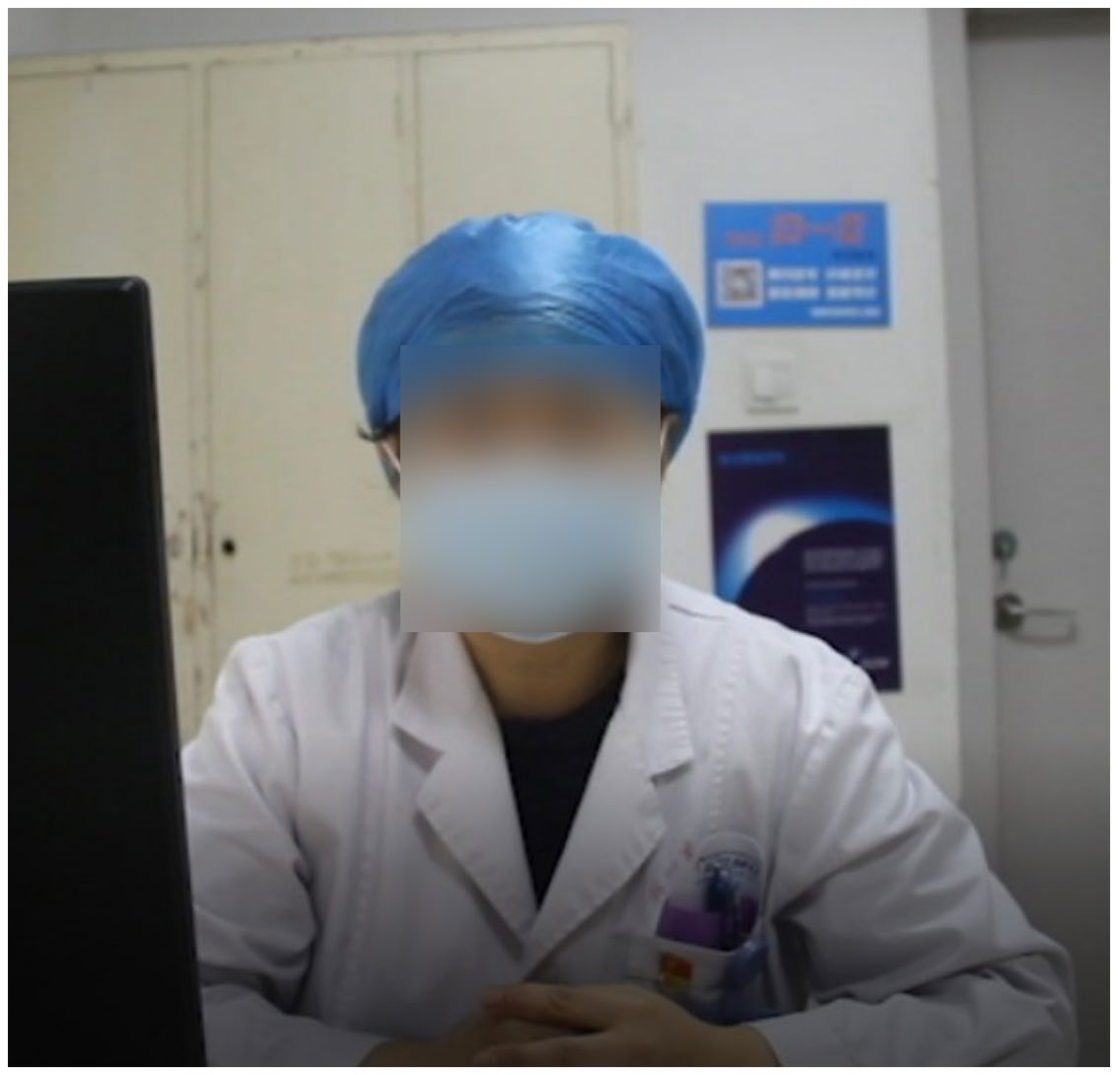
The video screenshot.

### Participants

Due to the lower incidence of breast cancer in males, it is more difficult for a male person to resonate with the disease itself. To avoid other emotional factors interfering with the results, we only took female samples for both experiments.

In the behavioral experiment, first, a total of 100 healthy medical students (mean age = 22) were recruited and 93 valid samples were collected due to missing values. Second, in cooperation with the oncology department of Central Hospital of Jin Zhou, a total of 15 breast cancer patients were recruited (mean age = 50) in two months. 6 of them are in stage II, 7 of them are in stage III. 2 of them are in stage IV.

In terms of EEG experiment, a total of 53 female students (mean age = 23) were recruited. All participants in both experiments understood the Chinese language, had communication experience with doctors, and had a self-reported normal or corrected vision. More specific information about participants is presented in the Table 3.

**Table 3:** Basic feature of participants.

Basic feature	Behavioral experiment					EEG experiment	
	93 Students			15 Patients		53 Students	
Age	Item	*n*	proportion	*n*	proportion	*n*	proportion
	<18	3	3.26%				
	18–25	86	93.48%			49	92.40%
	26–30	2	2.17%			4	7.50%
	31–40	1	1.09%				
	41–50			7	46%		
	51–60			6	40%		
	>60			2	13.30%		
Education							
	High school or below	1	1.09%	10	66.70%		
	Associate degree			3	20%		
	Bachelor degree	89	96.74%	2	13.30%	30	56.60%
	Master degree	1	1.09%			18	34%
	PHD degree	1	1.09%			5	9.40%

### Measures

#### Strategy and sceanrio

Five strategies were presented based on VR-CoDES. NRIa and ERIa stood for information delivery. EPAEm and NPIm stood for empathy. ERAb stood for authority. Six scenarios were presented as “Doubt,” “Fear of Death,” “Fear of the Future,” “Surgery,” “Hair Loss,” and “Upper Limb Disorders,” ranging from consultation, treatment, and “back to society” stage. Strategy and scenarios are measured as categorical data.

#### Emotion

Emotions were measured based on PANAS (Positive and Negative Affect Scale, PANAS; Watson et al., 1988). PANAS consists of two partial scales, PA (positive affect) and NA (negative affect), which include 10-item scales to measure both emotions. Each item is rated on a 5-point scale of 1 (not at all) to 5 (very much). Since some emotions in the PANAS scale were relatively rare in doctor-patient communication, we screened the emotion scale and added neutral emotions in line with the doctor-patient communication scenario. We divided emotions into two groups: positive emotions and negative emotions. There were four items for each group. The positive emotions were “satisfied,” “confidence,” “happy,” and “inspiration.” The negative emotions were “tense,” “worried,” “upset,” and “panic.” Each item was rated on a 7-point scale from 1 (totally not agreeable) to 7 (totally agreeable).

#### Trust

Trust was measured based on WFPTS (Wake Forest Physician Trust Scale; Hall et al., 2002). The WFPTS is a 10-item scale that consisted of five dimensions: fidelity, competence, honesty, confidentiality, and global trust. This study focused on doctor-patient communication in the context of bad news, so WFPTS was revised to a 4-item scale: “my doctor is extremely thorough and careful,” “my doctor’s medical skills are as good as they should be.,”” my doctor is totally honest in telling you about all of the different treatment options available for your condition,” and “All in all, I have complete trust in my doctor.” Each item was rated on a 7-point scale from 1 (totally not agreeable) to 7 (totally agreeable).

#### EEG measures

EEG data was recorded from 32 scalp sites with Ag/AgCI electrodes mounted in an elastic cap (Brain Products, Munich, Germany) according to the 10–20 international system. The bio-signals were recorded at a sampling rate of 500 Hz. The preparation procedure took about 10 min, during which time all electrodes were placed, and the DC offset of all sensors was kept below 20 μV. EEG data were collected for the entire duration of the experiment. All EEG data preprocessing was performed offline using custom Matlab code (Mathworks Inc., MATLAB, R2016b; EEGLAB, v14.1.1). As part of the preprocessing, the data was filtered by a high-pass filter to remove DC drifts. Subsequently, 45–55 Hz notch filters were applied to minimize the power-line noise interference, and all channels were then re-referenced to the average channel. The continuous EEG data was then epoch between 2000 ms before the movie trailer’s start time as a baseline amplitude. Due to video length, we divided each video into 5 segments. Each segment had 3,000 ms to preprocess conveniently. The eye-movement artifact was corrected using independent component analysis (ICA), as implemented in the EEGLAB toolbox. For each segment, we computed event-related spectral perturbation (ERSP; event-related shifts in the power spectrum). Time-frequency decomposition was determined using Morlet wavelets and the number of wavelet cycles was 1. To investigate EEG activities at different frequencies and times, we segmented data into the following frequency bands: delta (1-4hz), theta (4-8hz), alpha (8-12hz), beta (12.5-30hz) and time bands: 1 (0–6 s), 2 (6–13 s).

### Data collection procedure

In the behavioral experiment, all participants viewed a description of medical scenarios and were asked to imagine themselves in these scenarios as a patient. Participants were then asked to read patient lines, and physicians’ responses and indicate their feelings. All participants were compensated for their participation in the study.

**Figure 2:**
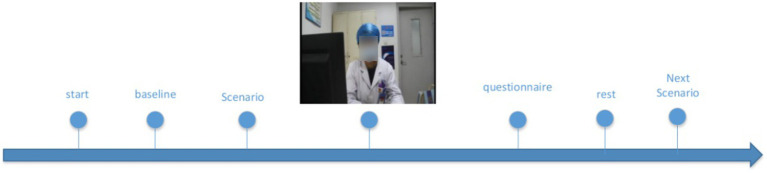
The process of EEG experiment.

In the EEG experiment, participants were seated in a comfortable chair and briefed on the objectives of the study. They were asked to watch a 5-min video about basic knowledge of breast cancer (such as “what is breast cancer?” or “what can cause breast cancer?”). Then participants were asked to answer a demographic questionnaire. Moreover, they were told that we would be collecting EEG and measures while watching those videos. Before the videos, participants observed a picture, in which a text version of all the scenario descriptions was shown, followed by an emotion and trust questionnaire as baseline data. Then the experiment started. Participants were asked to observe a text version of a scenario description. The presentation of the physician response video followed next. At the end of each response video, an on-screen questionnaire was presented asking the participants to report their emotional feelings and trust. 30 videos and scenarios were randomly presented. The frame rate of the video was 30hz, and the aspect ratio was 4:3. For all videos, the screen resolution was set to 720*540 and the sound was played through the speaker. The present study is carried out in accordance with ethical standards. A consent form was obtained from all participants before the experiment. The EEG process is presented in Figure 2.

### Data analysis

All data was analyzed using IBM SPSS Statistics (version 27.0). For all behavioral data, a repeated measure ANOVA method was conducted to investigate how physician response affected participant emotion and trust. Then, to explore optimal response strategy, multiple comparisons were used to compare the effects of different physician responses in the same medical scenario and the effects of the same physician’s response in a different medical scenario.

First, For EEG data, the repeat measured ANOVA method was applied to investigate how physician response affects EEG cluster activity in different medical scenarios and response strategies. The EEG activity was calculated by subtracting the baseline data measure for each frequency band.

Table 4 presents the results of relevant electrodes. The result shows a significant correlation between strategies and EEG activity on particular electrodes. Due to restrictions on space and clarity needs, we only present the significant *p*-values. We can find that the most significant scores are centralized in particular locations, like the frontal, parietal, and central parts of the cerebral cortex. Therefore, based on the result of ANOVA, for further analyses we aggregated the data of different frequencies and electrode locations, distinguishing the hemispheres as well as prefrontal, frontal, and parietal brain areas. Frontal delta (Fz), parietal delta (P3, P4, Pz), prefrontal theta (FP1, FP2), parietal theta (CPZ, P3, P4, Pz), frontal alpha (FC2, FC6, asymmetry), parietal alpha (CPZ, P3, P4, Pz), frontal beta (F3, F4, Fz, FC1), and central beta (C3, C4, CZ, CPZ). Aggregated data is averaged, then the ANOVA method is applied again. The aim of the ANOVA method in this part is to find locations of EEG electrodes and aggregate the data.

**Table 4:** Significant factors on EEG activity.

Electrode location	Scenario	Strategy	Scenario	Strategy	Scenario	Strategy	Scenario	Strategy	Scenario	Strategy	Scenario	Strategy	Scenario	Strategy	Scenario	Strategy
	Delta				Theta				Alpha				Beta			
	1		2		1		2		1		2		1		2	
P3						0.04**		0.03**				0.011**				
P4				0.042**			0.032**									
Pz				0.026**				0.026**				0.053*				
CPZ								0.013**	0.04**			0.017**			0.087*	0.037**
FP1	0.066*				0.002***				0.019**			0.028**		0.088*		0.085*
FP2				0.077*		0.018**				0.048**		0.092*				
FC2	0.042**							0.03**				0.073*	0.051*		0.021**	
FC6						0.01**			0.083*			0.009***	0.074*			0.059*
F3		0.08*														
F4																0.054*
Fz			0.032**	0.039**	0.053*							0.056*			0.019**	
FC1		0.049**							0.077*							0.079*
C3								0.05					0.009***		0.068*	0.056*
C4												0.079*	0.01**			
CZ	0.016**															

* Significant at the 0.1 level.

** Significant at the 0.05 level.

*** Significant at the 0.01 level.

Second, we applied a repeat-measured ANOVA method to aggregated data. The dependent variables are frontal delta (Fz), parietal delta (P3, P4, Pz), prefrontal theta (FP1, FP2), parietal theta (CPZ, P3, P4, Pz), frontal alpha (FC2, FC6, asymmetry), parietal alpha (CPZ, P3, P4, Pz), frontal beta (F3, F4, Fz, FC1), and central beta (C3, C4, CZ, CPZ). The independent variable is strategy, and the moderator is scenario. The aim of this step is to explore the correlation between EEG cluster activity and strategies.

Third, we conducted a stepwise regression method to select independent variables (EEG cluster activity) to be used in a final optimal model. Based on the result of stepwise regression, in terms of negative emotion, parietal alpha was selected as an independent variable. In terms of positive emotion, parietal theta and alpha, frontal and central beta were selected as independent variables. In terms of trust, frontal alpha was selected as an independent variable. The selection is shown in the appendix.

Finally, a GLM regression method was applied to investigate the relationship between selected EEG clusters’ activity, emotion, and trust. Through those methods for EEG data above, we intended to reveal the internal mechanism of immediate emotional response and explore the optimal communication strategy with EEG measurements objectively. All *p*-values were two-tailed, and *p*-values < 0.1 were considered statistically significant.

### Ethical approval

Ethical approval was obtained from the Research and Ethics Committee of the School of Business and Management, Shanghai International Studies University.

## Results

### Behavioral experiment

A 6 (scenario: Doubt, Fear of Death, Fear of the Future, Surgery, Hair Loss, Upper Limb Disorders)*5 (strategy: NRIa, NPIm, ERIa, ERAb, EPAEm) repeated measure ANOVA method on emotion and trust revealed a significant interaction between scenarios and strategy. Since Mauchly’s Test of Sphericity indicated that the assumption of sphericity had been violated (*p* < 0.1), corrected results were presented using the Greenhouse–Geisser method (Table 5).

**Table 5:** Factors contributing to negative, positive, and trust.

	Students (*n* = 93)				Patients (*n* = 15)			
	df	Mean square	*F*-value	*p*-value	df	Mean square	*F*-value	*p*-value
**Negative**								
Scenario	2.583	22.586	7.330	0.00024***	2.196	19.682	4.196	0.022**
Strategy	2.708	11.170	8.697	0.00003***	2.597	9.297	5.907	0.003***
Scenario* strategy	9.718	2.521	3.482	0.00019***	4.324	4.566	2.007	0.100
**Positive**								
Scenario	2.887	11.034	4.036	0.00867***	2.711	4.482	2.000	0.136
Strategy	2.690	15.059	10.248	0.00000***	1.626	51.648	7.951	0.004***
Scenario* strategy	9.812	2.995	3.606	0.00011***	5.001	4.139	3.057	0.015**
**Trust**								
Scenario	2.620	24.107	6.381	0.0010***	2.605	1.066	0.284	0.810
Strategy	2.634	207.286	86.504	0.0000***	2.259	51.497	9.548	0.000***
Scenario* strategy	11.303	17.077	19.508	0.0000***	3.507	12.875	3.895	0.011**

** Significant at the 0.05 level.

*** Significant at the 0.01 level.

In terms of medical student samples, it could be seen from Table 5 that the influence of scenarios on emotions (both negative and positive) is significant, which indicates that physicians’ responses in different scenarios can significantly affect participants’ emotions and trust. Significant results on strategy indicate that different communication strategies followed by physicians significantly affect participants’ emotions and trust. The interaction between scenarios and strategy is significant, which means that in the condition of a particular scenario, the different strategies followed by physicians can significantly affect emotions and trust. In terms of patient samples, it could be seen that the significant results are basically the same as student samples. Insignificant parts might be due to insufficient patient samples. Thus, we can compare the difference in communication strategy in each scenario through multiple comparisons.

Through multiple comparisons, we could compare the difference in communication strategies based on emotion and trust in each scenario, and then conclude the strategy matrix including effective strategies and avoiding strategies as shown in Table 6. The detailed information on multiple comparisons is shown in the appendix.

**Table 6:** The strategy matrix based on the ANOVA results.

Strategy	Effective			Avoiding		
	Negative Emotion	Positive Emotion	Trust	Negative Emotion	Positive Emotion	Trust
NRIa	Doubt, Fear of Death, Hair Loss	Hair Loss	Fear of Death		Surgery	
NPIm			Doubt		Doubt, Fear of Death, Hair Loss*	Fear of Death, Fear of Future, Surgery,Hair Loss*, Upper Limb Disorders*
ERIa	Surgery, Fear of Death, Upper Limb Disorders, Hair Loss*	Doubt, Fear of Future, Fear of Death, Hair Loss, Upper Limb Disorders, Surgery	Doubt, Surgery, Hair Loss, Upper Limb Disorders, Fear of Death*			
ERAb			Fear of Future	Doubt, Fear of Future, Fear of Death, Hair Loss, Upper Limb Disorders, Surgery	Doubt, Fear of Future, Hair Loss, Upper Limb Disorders	Doubt
EPAEm	Fear of Future, Hair Loss	Fear of Future, Hair Loss			Doubt	Doubt, Fear of Future, Hair Loss, Upper Limb Disorders

* Scenarios with * represents difference of results of patient samples.

The strategy matrix presents the scenarios in which effective and avoidable response strategies are applied based on emotion and trust. For example, physicians could apply NRIa, ERIa, or EPAEm if they want to mitigate patients’ negative emotions. Then they can choose strategies based on specific scenarios they are in. The results of student samples and patient samples are basically the same. Differences are marked with *. The detailed information is shown in the appendix.

### EEG experiment

A repeat measured ANOVA method was applied to investigate the relationship between scenarios, strategy, and EEG activity (Table 7).

**Table 7:** Effects of physicians’ response on EEG activity.

Segement	Source	Frontal Delta			Parietal delta			Prefrontal delta		
		Mean square	F	Sig.	Mean square	F	Sig.	Mean square	F	Sig.
1	Scenario	1.357	1.693	0.153	0.299	0.821	0.53	1.167	2.396	0.048^**^
	Rules	0.112	0.225	0.921	0.684	1.992	0.109	0.387	0.646	0.597
	Scenario * rules	0.734	0.897	0.55	0.726	1.262	0.234	0.762	0.798	0.631
2	Scenario	0.478	2.634	0.032^**^	0.074	0.456	0.779	0.391	1.533	0.189
	Rules	0.44	2.731	0.039^**^	0.238	1.665	0.169	0.285	1.381	0.249
	Scenario * rules	0.296	1.142	0.321	0.142	0.599	0.827	0.275	0.778	0.66
**Segement**	**Source**	**Parietal theta**			**Prefrontal alpha**			**Parietal alpha**		
		Mean square	F	Sig.	Mean square	F	Sig.	Mean square	F	Sig.
1	Scenario	0.346	1.028	0.392	0.899	1.307	0.269	0.407	0.695	0.582
	Rules	0.214	0.761	0.535	1.081	1.468	0.223	0.198	0.387	0.799
	Scenario * rules	0.524	1.041	0.407	0.613	0.641	0.785	0.55	0.813	0.638
2	Scenario	0.036	0.21	0.923	0.392	1.492	0.204	0.104	0.435	0.759
	Rules	0.33	2.444	0.077^**^	0.659	2.788	0.046^**^	0.687	3.583	0.021^**^
	Scenario * rules	0.156	0.719	0.678	0.391	1.223	0.271	0.233	0.878	0.547
**Segement**	**Source**	**Frontal alpha**			**Central beta**			**Frontal beta**		
		Mean square	F	*p*-value	Mean square	F	*p*-value	Mean square	F	*p*-value
1	Scenario	0.307	1.083	0.368	0.092	0.363	0.775	0.112	0.586	0.64
	Rules	0.189	0.531	0.694	0.467	2.274	0.075^*^	0.299	1.664	0.17
	Scenario * rules	0.522	1.171	0.296	0.446	1.121	0.348	0.313	0.846	0.544
2	Scenario	0.039	0.437	0.802	0.115	0.916	0.446	0.089	0.734	0.557
	Rules	0.259	3.667	0.008^***^	0.299	2.939	0.047^**^	0.307	3.133	0.037^**^
	Scenario * rules	0.098	0.842	0.611	0.168	0.911	0.495	0.172	0.951	0.464

* Significant at the 0.1 level.

** Significant at the 0.05 level.

*** Significant at the 0.01 level.

First, ANOVA on EEG clusters reveals that (1) most significant scores are from second time segment (6–13 s), which is logical because the most part of conversations take place in 6–13 s. Participants need some time to dive into the conversation. (2) there is a correlation between cluster EEG activity and strategies. Difference of communication strategy emerged with regard to cluster central beta (df = 3.33, *F* = 2.274, *p*-value = 0.075), frontal delta (df = 3.419, *F* = 2.731, *p*-value = 0.039), parietal theta (df = 2.526, *F* = 2.444, *p*-value = 0.077), prefrontal alpha (df = 2.844, *F* = 2.788, *p*-value = 0.046), parietal alpha (df = 2.844, F = 2.788, *p*-value = 0.046 < 0.05), frontal alpha (df = 3.786, *F* = 3.667, *p*-value = 0.008), central beta (df = 2.408, *F* = 2.939, *p*-value = 0.047), frontal beta (df = 2.475, *F* = 3.133, *p*-value = 0.037). The moderation of scenarios was not significant. Through multiple comparisons, we could explore which strategy correlated the cerebral activity more.

Second, through multiple comparisons in terms of each EEG activity, we aim to find which strategy has a greater correlation. This means that when such a strategy stimulates, the corresponding EEG activity may fluctuate more. Based on multiple comparison results, we can conclude that ERIa had a greater correlation with frontal delta activity compared to other strategies. NPIm had more correlation with frontal alpha, parietal alpha, central beta, frontal beta, and parietal theta activity than other strategies. EPAEm had a stronger correlation with prefrontal alpha activity than other strategies. The result above means information provision and empathy can relate to cerebral activity, and these cerebral activities were found through multiple comparisons.

Third, we use the GLM method to explain that the prefrontal theta and parietal alpha have significant effects on positive emotions while parietal alpha has a significant effect on negative emotions. Frontal alpha has a significant effect on trust. The result of this step explains the correlation between emotions and trust and these activities. Which means that when these emotions/trust occur, these EEG activities are active. The results are consistent with some previous research. The detailed information about multiple comparisons is shown in the appendix.

The GLM method was performed to investigate the effect of strategy on emotions/trust through cerebral activities. The result reveals that (1) most significant scores are from second time segment (6–13 s), which is logical because the most part of conversations take place in 6–13 s. Participants need some time to dive into the conversation. (2) As shown in Table 8, EEG activities in prefrontal theta, and parietal alpha have significant effects on positive emotions while parietal alpha activity has a significant effect on negative emotions. Frontal alpha activity has a significant effect on trust. From this result, we can conclude that information or empathy-related cerebral activities actually affect emotions and trust.

**Table 8:** EEG activity contributing to emotions and trust.

	Positive		Negative		Trust	
	Standardized coefficients	*p*-values	Standardized coefficients	*p*-values	Standardized coefficients	*p*-values
(Constant)		0.276		0.086		0.838
Subject	−0.137	0	0.187	0	−0.087	0.001
Scenario	−0.001	0.986	−0.023	0.694	0.011	0.848
Strategy	−0.03	0.6	0.047	0.399	0.028	0.624
S_R	0.11	0.152	−0.104	0.172	0.031	0.692
Parietal theta	0.045	0.305				
Central beta-1	−0.021	0.408				
Frontal beta-2	0.01	0.771				
Prefrontal theta-2	−0.091	0.003***				
Parietal alpha-2	0.085	0.043**	−0.073	0.003***		
Frontal alpha-2					0.061	0.015**
Adjusted R square	0.033		0.045		0.011	

** Significant at the 0.05 level.

*** Significant at the 0.01 level.

Fourth, combined with multiple comparison results, parietal alpha and frontal alpha have more correlation with strategy NPIm than others. The result shows a highly positive correlation between parietal alpha and positive emotion and a highly negative correlation between parietal alpha and negative emotion. It indicates that when alpha power increases, positive emotions increase and negative emotions decrease, which makes the parietal alpha a marker for moderate emotion. Similarly, in terms of trust, the result shows a high positive correlation between frontal alpha and trust. According to the correlation of strategy NPIm, physicians could adjust strategy NPIm to increase parietal alpha activity.

## Discussion

This research performs behavioral and EEG experiments to investigate the effect of physicians’ responses on patients’ emotions and trust through the first POV video stimuli and a communication strategy is presented in the context of breaking bad news. Distinguished from existing research, this study investigates the effect of responding strategies and explores the internal mechanism of presenting strategies through neuroscience experiments.

### Behavioral experiment

From it, we could conclude that physicians could apply strategy ERIa in most scenarios and strategy NRIa in many scenarios, which means information provision is significant when it comes to responding to patients’ cues and concerns. In delivering bad news, information offering could not only meet patients’ information needs but also ease their emotions. This finding is consistent with Ting et al. (2016) and Almyroudi et al.’s (2011) findings (53; 109). Through moderation of scenarios, we can conclude that physicians need to acquire not only medical knowledge but also knowledge about life and society, including moral values, common sense, and marriage values. These values matter equally to medical information. Compared to NRIa, ERIa is applied in more scenarios. It implies that physicians should respond clearly to patients’ cues and concerns.

The most important strategy that physicians need to avoid is ERAb. Refusing to respond to patients’ cues and concerns may cause their dislike. It indicates that patients prefer a patient-centered communication style, not doctor-centered, when it comes to responding to patients’ cues and concerns. This finding is consistent with the effective results of a “patient-centered” communication style (Cannon, 1927; Passik et al., 1995; Guo et al., 2013; Shu et al., 2018).

The controversial finding is the empathy strategy. In some scenarios (doubt, fear of death, fear of the future, surgery), implicit empathy strategies are not very effective compared to information giving, indicating that patients need more information instead of blindly empathizing and comforting. On the other hand, in some scenarios (Fear of the Future, hair loss), explicit empathy strategy plays an important role in responding compared to information-providing. Compared to the implicit empathy strategy, it implies that patients need an explicit response to their emotions. Doctors can focus on strategy selection based on needs (emotional focus or trust focus) to respond to patients.

### EEG experiment

This research conducts a video stimulus EEG experiment to test whether and how these strategies affect emotions and trust through EEG activities, find related EEG markers, and verify the affection through investigating the correlation between EEG markers and emotions and trust.

The research argued that parietal alpha power indicated arousal and intensity of emotional processing (Heller and Nitscke, 1997). Through an EEG experiment, we find the oscillations of parietal alpha power affect participants’ emotions. It indicates that the parietal alpha power has an emotional processing function, which is consistent with Heller and Nitschke’s findings. Then, through ANOVA results, we find parietal alpha power has more correlation with strategy NPIm than other strategies. It verifies that empathy strategy could actually affect patients’ emotions from a neuroscience perspective. Similarly, the result shows a high positive correlation between frontal alpha and trust, which is consistent with Oh et al.’s (2022) results. Frontal alpha power correlates with strategy NPIm more than other strategies. It verifies that an empathy strategy could actually affect patients’ trust from a neuroscience perspective, which is consistent with findings of previous research (Toutin-Dias et al., 2018; Mirza et al., 2019; Rasmus et al., 2020; Bukowski et al., 2021). Besides that, we find that when alpha power increases, positive emotions increase and negative emotions decrease, which makes the parietal alpha and frontal alpha markers moderate emotions and trust.

In a nutshell, in this post-pandemic age, the psychological treatment of patients is facing greater challenges and restrictions. The situation setting of delivering bad news strategy is restricted today, and existing strategies lack detailed patient emotion recognition and response methods. This research investigates the effects of response strategies (information delivery, empathy, and authority) on emotions and trust in hearing bad news and proposes a detailed strategy matrix, then verifies the effects of empathy on emotions and trust from a neuroscience perspective.

This study has the following pragmatic implications: (1) enhance the effectiveness of communication between doctors and patients. Improving the trust and emotions of patients lays the foundation for subsequent patients’ compliance with treatment. (2) Different video response strategies can be formulated according to different response strategies in different scenarios, and a video database can be established, which can be effectively applied to the mobile and PC terminal. According to the above strategy, doctors can provide video answers to patients’ questions in stages and scenes while answering questions, effectively comforting them, and effectively applying them to the online medical question and answer platform. (3) Instead of simply providing comfort, provide strategic guidance for online video conferences between doctors and patients. (4) Physicians could adjust strategies through EEG markers like parietal alpha and frontal alpha.

## Limitation

(1) Under the consideration of ethical problems, participants in the EEG experiment are students instead of actual cancer patients. (2) Due to the lower incidence of breast cancer in males, it is more difficult for a male person to resonate with the disease itself. To avoid other emotional factors interfering with the results, we only took female samples. Follow-up research can further investigate male samples. (3) Follow-up research can use the text of continuous dialogue between doctors and patients for verification. (4) The EEG frequency band can be studied in segments to verify the more detailed frequency band response. Follow-up research can be further verified to get accurate influencing factors. (5) Follow-up research can be focused on doctors’ facial expressions to comprehensively explore the influence of communication strategies on behavior. (6) follow-up research tend to combine CNS and ANS activity to detect patients’ emotion. (7) Due to the covid-19 and restrictions of the hospital, we could only take patient samples who were in the chemotherapy process. Many patients cannot be connected because they are at home. Therefore, the selected patients and students showed a great age difference. In future research, we tend to extend the sampling time and range of age.

## Data availability statement

The raw data supporting the conclusions of this article will be made available by the authors, without undue reservation.

## Ethics statement

The studies involving human participants were reviewed and approved by Research and Ethics Committee of the School of Business and Management, Shanghai International Studies University. The patients/participants provided their written informed consent to participate in this study.

## Author contributions

YS: conceptualization, methodology, and software. YX: data curation and writing-original draft preparation. FW: supervision, reviewing, and editing. WL: experimental materials editing and data resource. All authors contributed to the article and approved the submitted version.

## Conflict of interest

The authors declare that the research was conducted in the absence of any commercial or financial relationships that could be construed as a potential conflict of interest.

## Publisher’s note

All claims expressed in this article are solely those of the authors and do not necessarily represent those of their affiliated organizations, or those of the publisher, the editors and the reviewers. Any product that may be evaluated in this article, or claim that may be made by its manufacturer, is not guaranteed or endorsed by the publisher.

## Abbreviations

NRIa: Non-explicit reduce-space information advise
NPIm: Non-explicit provide-space implicit empathy
ERIa: Explicit reduce-space information advise
ERAb: Explicit reduce-space active blocking
EPAEm: Explicit provide-space empathy
VR-CoDES: Verona coding definitions of emotional sequences
